# Hypertension Secondary to Severe Aortic Coarctation in an Adult Patient

**DOI:** 10.7759/cureus.86128

**Published:** 2025-06-16

**Authors:** Victor Yair Gutiérrez Rangel, Martha Morelos Guzmán, Carlos Arturo Areán Martínez, Valeria Sandoval Martínez, Maria S. Fraga Ramos

**Affiliations:** 1 Medicine, Hospital General “Dr. Miguel Silva”, Morelia, MEX; 2 Cardiology and Cardiovascular Imaging, Hospital General “Dr. Miguel Silva”, Morelia, MEX; 3 Hemodynamics, Hospital General “Dr. Miguel Silva", Morelia, MEX; 4 Radiology and Cardiovascular Imaging, Hospital General “Dr. Miguel Silva”, Morelia, MEX

**Keywords:** computed tomography angiography, endovascular placement of stent, multimodalty cardiac imaging, secondary hypertension, severe coarctation of the aorta

## Abstract

A 42-year-old man with a 24-year history of poorly controlled hypertension was admitted following a traumatic femoral fracture. Cardiovascular evaluation was prompted by a 25 mmHg systolic blood pressure difference between the left upper limb (165/90 mmHg) and the left lower limb (140/85 mmHg), along with an interscapular systolic murmur, during trauma admission, suggesting coarctation. Chest X-ray revealed rib notching and the classic “3-sign,” raising suspicion of aortic coarctation. Transthoracic echocardiography (TTE) showed no intracardiac deformity and demonstrated a peak-to-peak gradient of 60 mmHg across the aortic isthmus. CT angiography (CTA) confirmed a severe coarctation, with a minimal luminal diameter of 3 mm, located 38 mm distal to the left subclavian artery, and extensive collateral circulation via intercostal and internal mammary arteries. A staged endovascular approach was undertaken due to complex anatomy, with successful deployment of two BeGraft covered stents (24×48 mm and 22×48 mm). Despite anatomical correction, the patient remained hypertensive, was discharged 10 days later without symptoms, and was enrolled in regular follow-up for blood pressure control.

## Introduction

Coarctation of the aorta (CoA) is a rare cause of secondary hypertension, accounting for <1% of cases. It may remain undiagnosed for years due to subtle clinical signs [1]. Defined as a narrowing of the aorta is one of the most common congenital heart defects, accounting for 4% to 6% of these diseases, with an incidence of up to four cases per 10,000 live births, occurring 1.7 times more in males than females [2]. CoA can present as a discrete stenosis or a long, hypoplastic aortic arch segment, typically at the ductus arteriosus insertion. Also, rare ectopic cases may occur in the ascending, descending, or abdominal aorta [3].

The most widely accepted pathophysiological mechanism involves the postnatal proliferation of ductal tissue within the aortic wall following exposure to arterial oxygen [4]. CoA is anatomically classified as preductal (proximal to the ductus arteriosus) and postductal (distal) types, with postductal being more common in older children and adults [2]. About 60% of CoA patients have a bicuspid aortic valve [5]. Other associated anomalies relevant in adults include aortic arch hypoplasia (18%) and genetic conditions such as Turner syndrome. Extracardiac anomalies may include aberrant right subclavian artery origin, intercostal collateral circulation, and intracranial aneurysms [4].

Clinically, milder forms of coarctation may remain asymptomatic until adulthood. When symptoms appear, they often reflect the hemodynamic consequences of the aortic narrowing. Proximal to the coarctation, elevated systolic pressure can lead to left ventricular overload, resulting in headache, epistaxis, and upper extremity hypertension. Distally, reduced blood flow contributes to exertional dyspnea, chronic fatigue, intermittent claudication, and, in more advanced cases, syncope. Key physical findings include a systolic blood pressure gradient greater than 20 mmHg between the upper and lower limbs, diminished or delayed femoral pulses, and systolic or continuous murmurs audible over the interscapular region [1].

Although chest radiography has limited sensitivity and specificity, it may suggest CoA through findings such as the "3 sign," rib notching between the third and ninth ribs, and ascending aortic dilation. Postmortem studies have shown rib notching in 75% of cases, with recent reports showing detection rates of 64%-68% [6]. Guidelines suggest transthoracic echocardiography (TTE) as the preferred initial diagnostic tool for identifying CoA, assessing pressure gradients, and detecting associated anomalies [3]. However, although TTE is inexpensive, rapid, and readily available, imaging adults can be challenging due to suboptimal echocardiographic windows, and it is also limited in its ability to visualize the aortic arch and proximal descending aorta [7].

Cardiac magnetic resonance (CMR) is the gold standard for evaluating congenital heart disease in adults, with a sensitivity of 86% and specificity of 100% for this disease. It precisely localizes coarctation, quantifies pressure gradients, assesses collateral flow, and identifies other anomalies [8]. CT offers sensitivity over 95%, providing high-resolution anatomical detail of the aorta, aortic valve, and coronary arteries [9]. CT angiography (CTA) is preferred for pre-interventional planning, thanks to its short acquisition time and exceptional spatial resolution, aided by advanced techniques like multiplanar reformats and volume rendering. These enable thorough evaluation of coarctation severity, vessel relationships, access routes, and collateral networks. Additionally, head CTA screens for intracranial aneurysms, found in up to 5% of CoA cases, linked to higher mortality [10].

According to guidelines, European Society of Cardiology (ESC) 2024 and European Association for Cardio-Thoracic Surgery/Society of Thoracic Surgeons (EACTS/STS) 2024 recommend definitive repair for any clinically significant CoA (Class I, Level C). ESC guidelines specify surgical or endovascular repair for hypertensive patients with an invasive peak-to-peak gradient > 20 mm Hg. They also favor endovascular intervention (Class IIa, Level C) for ≥ 50% aortic narrowing, even with a gradient < 20 mm Hg, and for normotensive patients with an invasively confirmed noninvasive gradient > 20 mm Hg [1]. EACTS/STS recommends intervention (Class IIa, Level C) in asymptomatic adults with a resting gradient > 20 mm Hg or a > 10 mm Hg difference in systolic pressure between upper and lower limbs, as well as in hypertensive adults with > 50% luminal narrowing, regardless of gradient. Additionally, referral to a specialized aortic center and consideration for endovascular repair are recommended for anatomically suitable patients (Class IIa, Level C) [10].

Traditionally, surgery has been the gold standard, with 10-year survival rates of 93%-98%. However, endovascular stent placement has recently become first-line therapy in high-risk adults or those with suitable anatomy [11,12]. Medical therapy is reserved for patients with mild CoA and no significant hypertension or organ damage. Treatment includes beta-blockers, angiotensin-converting enzyme (ACE) inhibitors, or angiotensin receptor blockers (ARBs), and regular imaging follow-up [1].

The prognosis for untreated aortic coarctation in adults is poor; historical data indicate a mean age of death at 34 years, with 75% of patients deceased by age 43, primarily due to heart failure, aortic dissection or rupture, endocarditis, and intracranial hemorrhage [13]. This case underscores the necessity of considering this diagnosis in older adults, particularly those with unexplained or resistant hypertension.

## Case presentation

A 42-year-old male patient with a medical history of systemic arterial hypertension diagnosed at age 18 and poor adherence to pharmacological treatment was admitted to the emergency department following a motor vehicle accident, resulting in an open fracture of the right femur. During the admission, resistant hypertension despite initial treatment, coupled with suggestive physical findings, raised suspicion of CoA, which was later confirmed as an incidental diagnosis through trauma imaging, revealing findings consistent with congenital heart disease.

On physical examination, heart rate was 88 bpm, blood pressure 199/116 mmHg (measured in the left upper limb), respiratory rate 18, temperature 37°C, and SpO_2_ 97%. The patient reported intermittent headaches consistent with severe hypertension. A prominent carotid pulse was noted bilaterally. A grade III/VI holosystolic murmur radiating from the left upper sternal border was auscultated, accompanied by a continuous murmur in the interscapular region. Lower extremity pulses were diminished, with weak femoral pulses bilaterally and a 25 mmHg systolic pressure differential; the left lower limb showed a pressure of 174/90 mmHg, indicating a significant gradient consistent with severe coarctation. The lower limbs were noted to be slightly cooler to the touch compared to the upper limbs, and no evidence of atrophy or cyanosis was observed.

**Figure 1:**
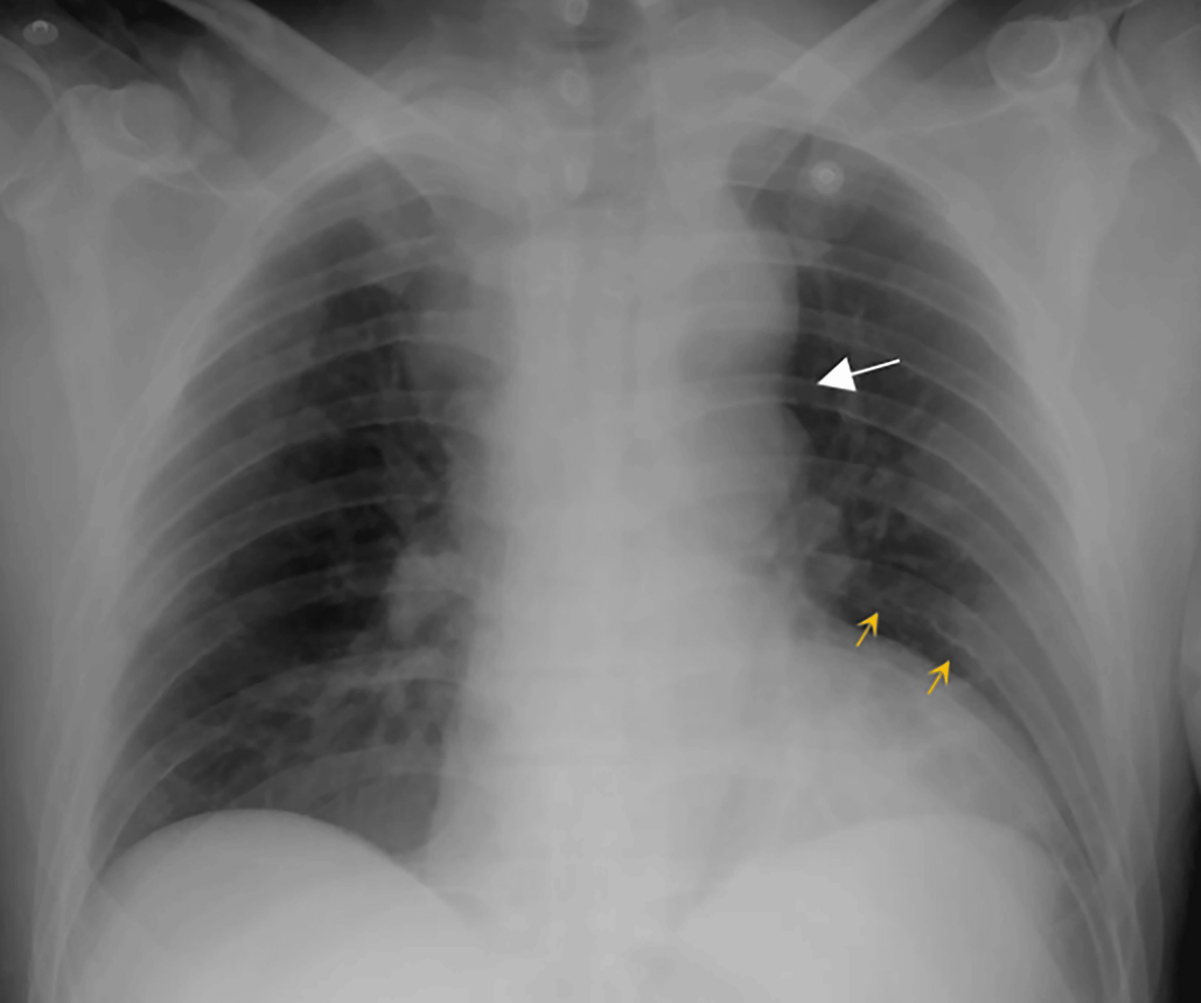
Posteroanterior chest radiograph showing the “figure 3 sign,” characterized by an indentation along the contour of the thoracic aorta due to a focal coarctation with pre and poststenotic dilatation (white arrow). Rib notching is also noted bilaterally (yellow arrows), representing collateral circulation through the intercostal arteries. This finding, known as “Roesler’s sign,” is caused by erosion of the inferior borders of the ribs secondary to increased flow through enlarged collateral vessels.

**Figure 2:**
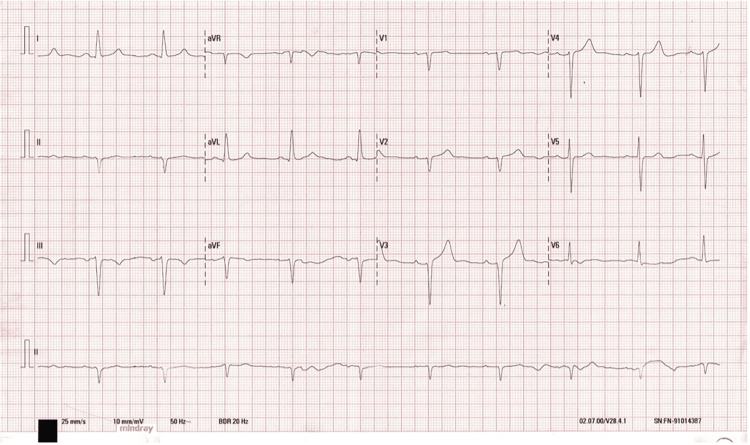
Electrocardiogram showing sinus rhythm at 88 bpm, left axis deviation (–30°), QRS duration of 120 ms, and rS complexes in leads II, III, and aVF.

**Figure 3:**
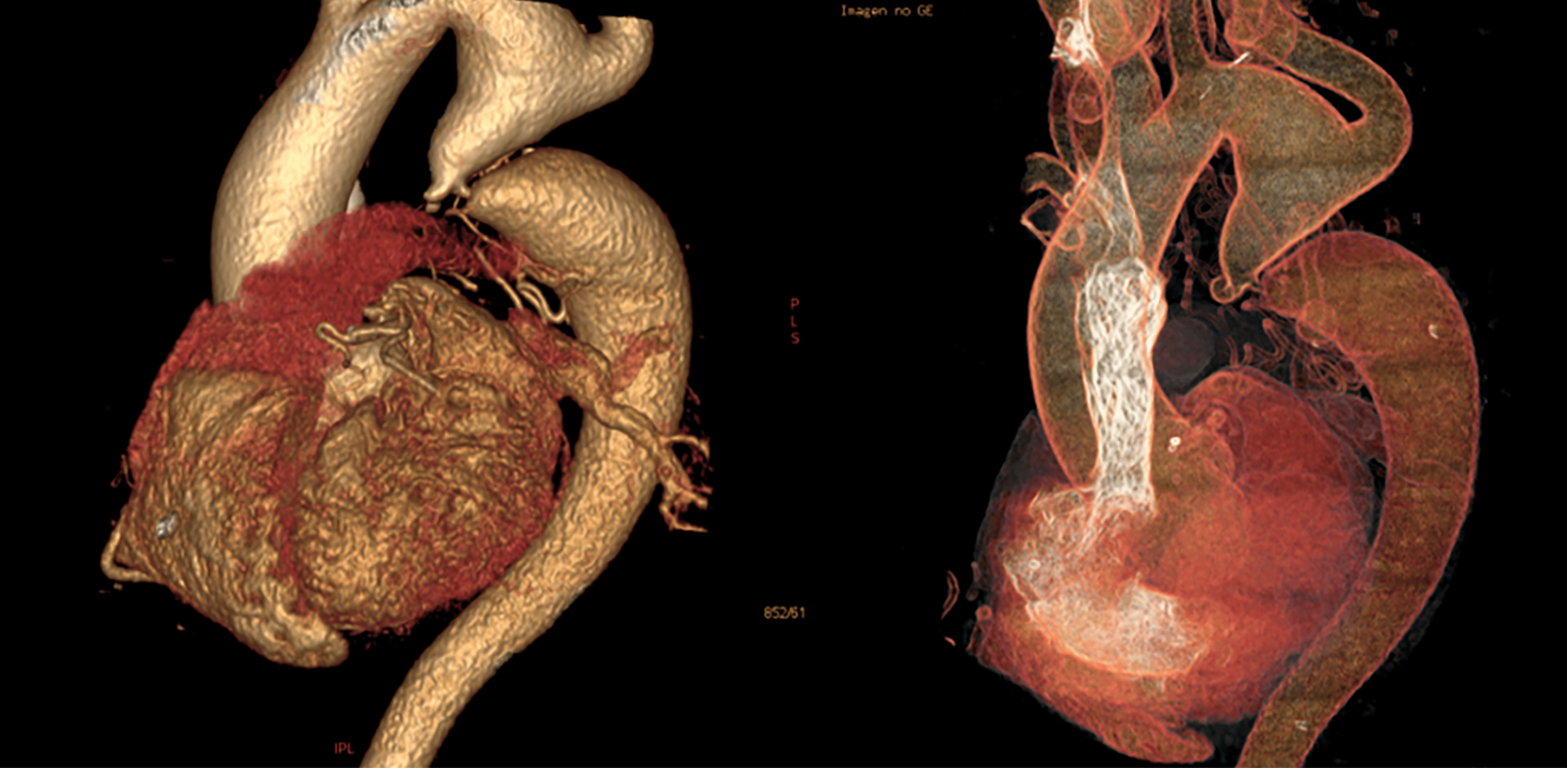
Three-dimensional reconstructions from aortic CTA demonstrating a postductal aortic coarctation with significant dilatation of the aortic segments proximal and distal to the narrowing. CTA: CT angiography

**Figure 4:**
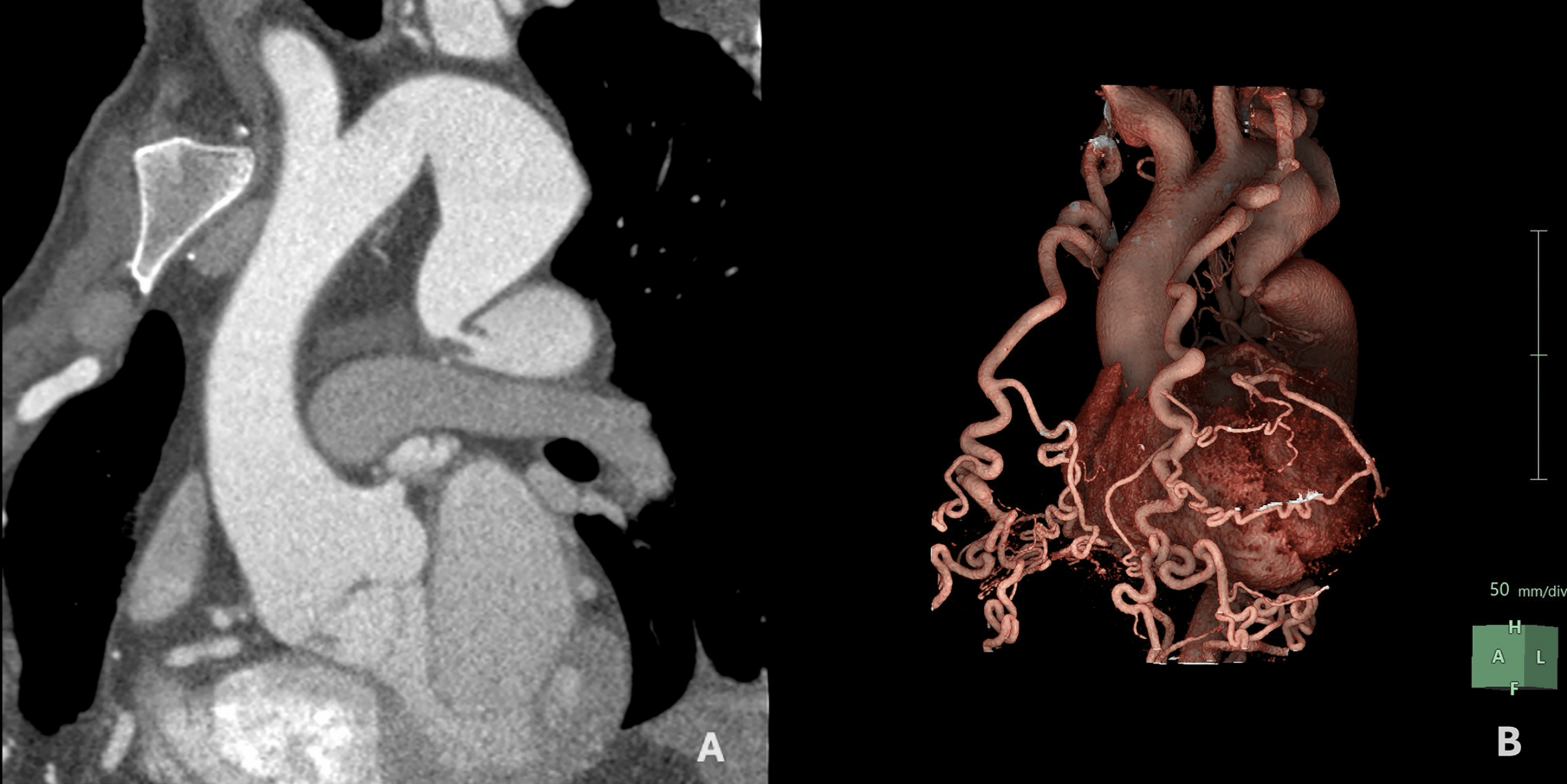
(A) Curved planar reformation of aortic CTA showing a severe postductal aortic coarctation with a minimal luminal diameter of 3 mm. (B) Volume-rendered reconstruction demonstrating marked dilation of both internal mammary arteries CTA: CT angiography

Routine blood biochemistry was within normal limits. Chest radiography revealed the “3 sign” and rib notching (Figure 1). Electrocardiography (ECG) showed sinus rhythm with left anterior fascicular block, a finding often associated with long-standing left ventricular pressure overload. No signs of ventricular hypertrophy or repolarization abnormalities were observed (Figure 2). Thoracoabdominal CTA demonstrated a tricuspid aortic valve and a severe postductal aortic coarctation with a minimum luminal diameter of 3 mm, located 38 mm distal to the origin of the left subclavian artery, and a post-stenotic segment measuring 34 mm in diameter. There was prominent collateral circulation involving the intercostal, internal mammary, and vertebral arteries (Figures 3, 4). Assessment of left ventricular systolic function using contrast-enhanced CT with volumetric analysis showed preserved function, with an estimated ejection fraction of 62%, which is consistent with a favorable prognostic profile in patients with CoA.

The patient’s antihypertensive regimen was modified to include enalapril 5 mg twice daily, metoprolol 50 mg once daily, and amlodipine 10 mg once daily, targeting afterload reduction, heart rate control, and vasodilation to stabilize hemodynamics. Following a multidisciplinary assessment, orthopedic surgical repair of the femoral fracture was prioritized and completed before cardiac intervention, with no intraoperative or perioperative cardiovascular issues reported, given stable hemodynamic status. Subsequent referral was made to the interventional cardiology unit.

**Figure 5:**
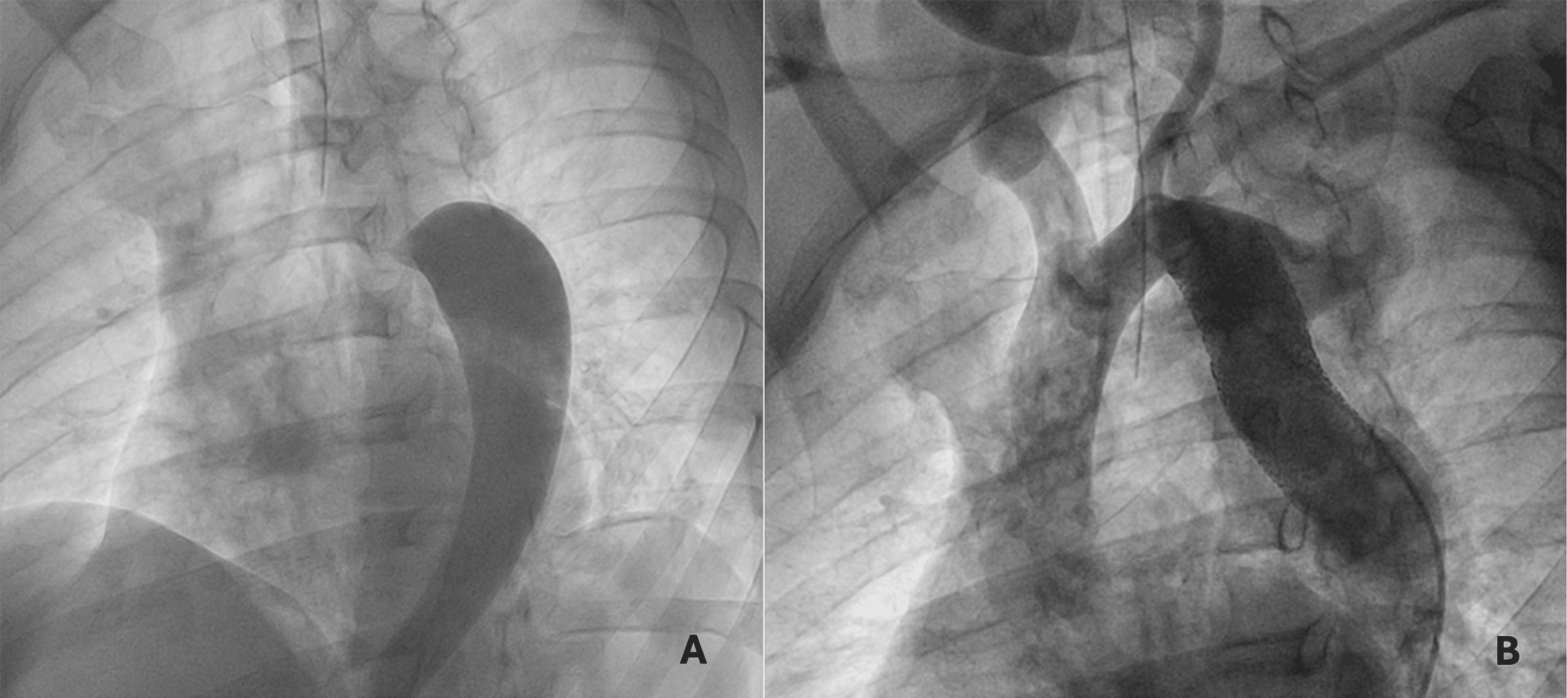
(A) Pre-intervention thoracic aortogram showing severe aortic coarctation with limited contrast passage across the narrowed segment. The minimum luminal diameter at the coarctation site measured 3.6 mm. (B) Post-intervention aortogram demonstrating successful dilation of the coarcted segment with satisfactory contrast flow; the final luminal diameter reached 16 mm after stent placement.

Cardiac catheterization revealed no coronary artery disease and confirmed a severe postductal aortic coarctation with minimal contrast passage (Figure 5). Access was obtained via the right common femoral artery utilizing a 6 Fr sheath system. Hemodynamic assessment indicated a peak-to-peak systolic gradient of 60 mm Hg. Left anterior oblique angiography provided measurements of the aortic diameters: 28 mm at the level of the transverse arch, 3.6 mm at the coarctation site (minimum lumen), and 28 mm immediately distal to the stenosis. Due to the complex anatomy of the coarctation, a staged endovascular strategy was employed, beginning with the deployment of a 24×48 mm BeGraft covered stent, however, residual narrowing and incomplete correction of the curvature persisted, prompting the implantation of a second stent (22×48 mm) to restore the aortic lumen and provide adequate radial strength. Post-procedural measurements showed a final luminal diameter of 16 mm at the treated segment, with resolution of the trans-stenotic pressure gradient. The procedure was completed without complications. Follow-up imaging demonstrated full restoration of aortic lumen integrity.

After endovascular intervention, the patient remained hypertensive, an expected outcome due to long-standing vascular remodeling, with further management steps planned, including optimization of antihypertensive therapy and regular follow-up. After achieving clinical stability and a thorough reassessment, he was discharged in good general condition. 

Long-term follow-up began four weeks after discharge, scheduled at 3, 6, and 12 months in the first year, then annually. Each visit included a comprehensive clinical assessment with four-limb blood pressure measurement, ECG, and chest X-ray. To evaluate stent position, aortic wall integrity, and rule out complications like aneurysm formation, a contrast-enhanced CTA was scheduled six months post-procedure. 

## Discussion

In this case, CoA remained undetected for over two decades, initially misdiagnosed as essential hypertension. The delayed diagnosis in this case underscores missed opportunities for early detection, such as routine blood pressure measurements in all four limbs and careful auscultation of the thoracic murmur, potentially detectable even in asymptomatic adults. 

Similar to the case reported by Cicek et al., involving a 52-year-old patient who remained virtually asymptomatic until his fifth decade of life, our patient exemplifies how extensive collateral circulation can effectively mask clinical signs of coarctation [14]. In contrast, Meller et al. described a case of severe adult-onset CoA presenting with refractory hypertension, highlighting a different compensatory pattern [15]. Our patient, who presented with resistant hypertension despite being previously diagnosed with essential hypertension, represents a middle ground between these two scenarios. The delayed diagnosis well into adulthood is atypical, as CoA is usually identified during childhood or adolescence. Collectively, these cases, including ours, underscore how aortic coarctation may go unrecognized for years, especially when hemodynamic obstruction is mitigated by robust collateral flow. Resistant hypertension, as seen in our patient, remains the most frequent clinical manifestation in adults with uncorrected CoA who otherwise may remain clinically silent [15].

While CT and MR angiography are the diagnostic gold standards, their availability remains limited in certain resource-constrained settings. In such contexts, doppler echocardiography, suprasternal views, and clinical scoring systems based on pulse differentials or upper/lower limb pressure gradients can serve as useful initial screening tools. Advanced imaging modalities were fundamental to achieving an accurate diagnosis

Hemodynamic evaluation via catheterization revealed a peak-to-peak systolic pressure gradient of 60 mmHg, providing objective confirmation of the obstruction’s severity. The presence of extensive collateral circulation, responsible for the characteristic “3 sign” and rib notching on imaging, reflects the chronic development of alternative flow pathways through the intercostal, internal mammary, and vertebral arteries. These findings are consistent with prior series in which more than 60% of adult patients with CoA showed radiologic evidence of intercostal collaterals [16].

The use of covered stents in native aortic coarctation in adults is well supported by contemporary literature, as they offer greater procedural safety by reducing the risk of aortic wall complications, such as dissection or rupture, compared to bare-metal stents or balloon angioplasty alone. Multicenter studies, including COAST II, have demonstrated near 100% technical success rates and significant reductions in transcoarctation gradients, along with improved blood pressure control in most patients [17]. In the present case, due to the complex anatomy characterized by severe narrowing and persistent curvature, a staged endovascular approach was adopted using two BeGraft covered stents (24×48 mm and 22×48 mm). Following deployment of the first stent, residual luminal narrowing and incomplete correction of the aortic curvature were observed, and full luminal restitution was not achieved, prompting the implantation of a second overlapping stent. This double-layer technique, selected in response to the patient’s complex anatomical features, enhances radial strength, improves wall apposition, and minimizes the risk of stent collapse or deformation. The sequential implantation of two endoprostheses achieved complete anatomical correction, restored the aortic lumen to 16 mm, and fully resolved the trans-stenotic pressure gradient, following current recommendations and recent clinical evidence [18].

Despite the immediate hemodynamic benefits of endovascular repair, residual hypertension is a well-documented concern in adult patients. Persistent hypertension after CoA repair is attributed to chronic vascular remodeling, characterized by increased arterial stiffness and baroreceptor resetting due to prolonged pressure overload. This likely triggered sustained neurohormonal activation, contributing to the need for intensified antihypertensive therapy in our patient. Studies reveal that up to two-thirds of individuals remain hypertensive post-intervention, which may be attributed to these pathophysiologic changes [13]. The need for intensified antihypertensive therapy in our patient highlights the relevance of individualized pharmacologic strategies and sustained monitoring to mitigate the risk of long-term cardiovascular morbidity.

Our follow-up strategy in this case report is closely aligned with the recommendations outlined by the ESC. According to the 2020 ESC Guidelines, which recommend annual clinical evaluations and serial cross-sectional imaging every three to five years, we arranged outpatient assessments at 3, 6, and 12 months during the first year, followed by yearly visits thereafter [2]. Each visit included a thorough clinical examination, bilateral blood pressure measurements, ECG, and chest radiography. In addition, a CTA was performed at six months to evaluate stent patency and rule out early complications. CTA was selected over CMR due to its accessibility and practical application within our institution.

## Conclusions

CoA can be a significant contributor to secondary hypertension, particularly in younger patients who experience resistant hypertension despite receiving optimal medical treatment. This case illustrates that CoA may not be confined to childhood; it can remain undetected for many years into adulthood due to nonspecific clinical signs or the body's ability to compensate through collateral circulation.

A thorough physical examination, which includes measuring blood pressure in all four limbs and assessing pulse quality, is essential for maintaining a high suspicion index for diagnosis. However, early confirmation typically requires imaging studies. In this patient's case, a CTA was pivotal for procedural planning and short-term monitoring, while cardiac MRI is recommended for long-term surveillance. The patient underwent successful staged stenting, achieving complete anatomical correction. Nevertheless, hypertension persisted, necessitating an increase in antihypertensive medication. This underscores the importance of personalized medical management and the need for regular follow-ups to monitor for late complications such as restenosis or aneurysm formation. Early detection and a multidisciplinary approach can significantly improve optimal outcomes for adults with CoA
